# Dynamic Permittivity Measurement of Ground-Tire Rubber (GTR) during Microwave-Assisted Devulcanization

**DOI:** 10.3390/polym14173543

**Published:** 2022-08-29

**Authors:** Rafael Pérez-Campos, José Fayos-Fernández, Juan Monzó-Cabrera, Fernando Martín Salamanca, Juan López Valentín, José Manuel Catalá-Civera, Pedro Plaza-González, Juan Rafael Sánchez-Marín

**Affiliations:** 1 Departamento de Tecnologías de la Información y las Comunicaciones, Universidad Politécnica de Cartagena (UPCT), 30202 Cartagena, Spain; 2 Instituto de Ciencia y Tecnología de Polímeros (ICTP-CSIC), Juan de la Cierva 3, 28006 Madrid, Spain; 3 Instituto de Tecnologías de la Información y las Comunicaciones, Universitat Politècnica de València (UPV), 46022 Valencia, Spain

**Keywords:** ground tire rubber, devulcanization, microwave, dielectric heating, permittivity, resonant technique, dielectric constant, loss factor, time-domain NMR

## Abstract

Many efforts are being made to find innovative ways of recycling rubber from end-of-life tires (ELTs), also called ground tire Rubber (GTR). Recycling through devulcanization allows the reintroduction of rubber back into the manufacturing industry. Such a process requires providing enough energy to break the sulfur links, while preventing damage to the polymeric chain. Microwave heating is controllable, efficient, and it does not rely on conventional heating mechanisms (conduction, convection) which may involve high heating losses, but rather on direct dielectric heating. However, to adequately control the microwave-assisted devulcanization performance, a thorough knowledge of the GTR permittivity versus temperature is required. In this work, GTR permittivity was monitored during its devulcanization. A resonant technique based on a dual-mode cylindrical cavity was used to simultaneously heat rubber and measure its permittivity at around 2 GHz. The results show sharp changes in the GTR permittivity at 160 and 190 °C. After the GTR cooled down, a shifted permittivity evidences a change in the GTR structure caused by the devulcanization process. Microwave-assisted devulcanization effectiveness is proven through time-domain nuclear magnetic resonance (NMR) measurements, by verifying the decrease in the cross-link density of processed GTR samples compared to the original sample.

## 1. Introduction

Nowadays, the storage capabilities for the disposal of end-of-life tires (ELT) are surpassed due to the slow rate of decomposition and insufficient recovery flowrate of their materials. Their management is an environmental challenge worldwide, as stated in [1,2]. In 2017, more than 29 Mt of ELTs were produced among 45 countries around the world (3.45 Mt within Europe), with a recovery efficiency of 90% [3,4].

It is well known that the recycling process is a far better solution than the energy recovered from combustion processes or landfill accumulation in terms of environmental impact and sustainability. Through recycling, it is possible to recover around 74% of the material from an ELT in the form of rubber granulate, also known as ground tire rubber (GTR), and several innovative ways for reusing and recycling GTR have been explored [1,2,3,4].

The main composition of GTR (44%) is a mixture of rubber matrices that have been vulcanized [5] to consolidate a thermostable polymer against deformation due to heat. Given that the worldwide rubber (natural and synthetic) consumption has been increasing at a rate of around 800 kt/year, reaching more than 29 Mt in 2018 (3.7 Mt within Europe) [3], recycled rubber from GTR is of great interest to contribute to a better environment, preserve the limited natural feedstock, and cover a portion of the rubber demand while reducing non-rubber producing countries’ reliance on imports.

Nevertheless, the consideration of GTR as a valuable rubber feedstock for the manufacturing industry relies on its ability to be processed to produce rubber products, thermoplastic elastomers, and blends in composite materials [5], and the most suitable way is through its devulcanization [6]. Rubber is originally a polymer, which achieves its unique elastomeric properties after the vulcanization process, where the polymer chains are cross-linked by the complex reaction of sulfur, activators, and accelerants with the double bonds of the rubber backbone. Tires are complex products made of different vulcanized rubber matrices mixed with around 24% of fillers (carbon black and silica), and other additives (processing oils, antioxidants, waxes, etc.) to build up their required performance. Devulcanization, therefore, entails sulfidic cross-links being broken to increase flexibility and decrease elasticity in the devulcanized GTR, enabling rubber to be processed again.

Devulcanization requires either physical, thermal, mechanical [7], microwave [8], ultrasonic [9], biological [10], or chemical [11] processing. It is of utmost importance to develop technologically feasible and cost-effective methods for recycling GTR, which could be a combination of these approaches, in order to achieve high devulcanization efficiency and selectivity. In terms of devulcanization uniformity, only microwave and ultrasonic methods can instantly heat the entire GTR particle, whereas others heat the material from its material surface to the particle’s core. In addition, mechanical, microwave, and ultrasonic technologies allow the modulation of the power being transferred at any time, providing full control of the process. The other methods imply a deposition of the whole energy into the system, and the power transference is not stoppable until it has been depleted fully.

Microwave-assisted devulcanization may provide more uniform heating than traditional heating methods based on heat convection and/or conduction [12]. This type of devulcanization relies on volumetric heating of the material by microwaves. Given the possibility of obtaining good mechanical and chemical properties of devulcanized material, understood as a quality standard that allows devulcanized rubber to be reintroduced in production processes, and the possibility of high productivity [13], microwave devulcanization is currently one of the most promising techniques. This process is classified as thermal devulcanization, and it does not involve chemicals during the process. Thus, this type of devulcanization is considered an eco-friendly technology at present [14]. However, the parameters governing the process must be engineered and thoroughly controlled to avoid degradation of the material due to excessive processing temperatures or uneven devulcanization degrees.

The energy efficiency and temperature distribution of microwave devulcanization greatly depend on the complex relative electric permittivity ($\varepsilon_{r}=\varepsilon_{r}^{\prime }-j\varepsilon_{r}^{\prime \prime }\;$) of rubber and, thus, a thorough understanding of such dielectric properties (dielectric constant $\varepsilon_{r}^{\prime }$ and loss factor $\varepsilon_{r}^{\prime \prime }$) becomes extremely important. The determination of dielectric properties for every kind of rubber has been a widely researched issue for many years in microwave applications [15,16,17,18,19,20]. Elastomers, such as natural rubber (NR) [15,16], styrene–butadiene rubber (SBR) [15], and nitrile–butadiene rubber (NBR) [15] have a low microwave absorption capacity (low $\varepsilon_{r}^{\prime \prime }$). This limitation can be overcome by the addition of a conductive filler, such as carbon black [13]. Other rubber-based compounds have also been studied to determine their permittivity behavior. For instance, ferrite rubber [17], rubber sheets containing carbon particles and carbon fiber [18], and iron particles disposed in a rubber matrix [19], as well as some individual rubber compound constituents have been examined [20].

The frequency range is, generally, one of the main aspects regarding dielectric measurements of materials. There are several studies at 1 GHz [19], 8.5–10.5 GHz [18], 1–12 GHz [16], 8–12 GHz [17], 5–24 GHz [20], and up to 26 GHz [15].

While some types of rubber do not perform well in dielectric heating when exposed to microwaves [21], GTR heats significantly due to the presence of carbon black that is mixed with the rubber. In this case, carbon black acts as an electromagnetic susceptor by absorbing the microwave energy and dissipating it into thermal energy. The speed of this energy transduction may increase with the rise of the temperature, so much so that if the power is not delivered conveniently, it could lead to a thermal runaway, causing the breakage of not only of the sulfidic bonds but also the polymeric chains that should be preserved. In order to prevent rubber carbonization caused by a thermal runaway, knowledge of GTR permittivity dynamics during the devulcanization process is fundamental.

Several studies have been conducted to find out the dielectric properties of GTR mixed with other composites [22,23,24]. Such studies reveal permittivity values for those mixtures at temperatures ranging from 20 °C to 120 °C [22,23], approximately, and also for a constant temperature [24]. To the best of the authors’ knowledge, there is not any publication that simultaneously devulcanizes GTR while measuring its dielectric characteristics. To achieve a deeper understanding of GTR permittivity evolution while devulcanizing, various temperature profiles and microwave power regimes have been applied. Hence, in this work, we present dynamic permittivity measurements for granulated GTR, the results of which are useful to adequately design devulcanization processes assisted by microwaves.

There are many techniques for measuring the permittivity of materials. Available techniques can be classified as resonant or non-resonant [25]. Resonant methods can also be subdivided into resonant cavities [26], open resonators [27], and dielectric resonators [28].

Additionally, different transmission lines are used for implementing sensing electromagnetic structures. Coaxial, waveguide, and microstrip lines are common methods for implementing the measuring circuit [29]. Some of these techniques are based on open configurations; that is, part of the energy is radiated out to the environment (e.g., free-space measurements, coaxial probes, or microstrip lines). Others are based on closed configurations; that is, the energy is confined inside the measurement device (e.g., a waveguide setup). Another classification can be made according to the number of measuring ports used (one or two), that is, measuring just the reflected wave or measuring both the reflection and the transmission [29].

Sometimes, the handling of the material to make it fit exactly into the sample holder dimensions requires some adaptation that could even lead to its destruction. Consequently, techniques can also be divided into destructive or non-destructive methods.

The choice of the most suitable method, or a combination of them, depends on the frequency of interest, the expected electrical losses, the amount of available material, its homogeneity, shape, and adaptability to the sample holder, the accuracy required, and whether the technique is required for just research or routine measurements. If the study must be carried out by varying the temperature, the existing techniques must be adapted for that purpose, and some techniques are more suitable to meet this requirement than others. Techniques based on the Nicholson–Ross–Weir method were discarded because they require a closed waveguide setup, which makes sample holding and temperature sensing difficult. The open coaxial probe technique was also discarded because of the materials’ form, since its measurements would have been inaccurate due to the presence of air gaps between the granules.

In this work, the experimental setup and methodology described in [30] for the GTR dynamic permittivity measurements were used. The obtained results show that, as a general trend, the permittivity of GTR increases when the temperature rises. Furthermore, the permittivity values of GTR after being heated are different from the ones measured before the devulcanization process occurs. This implies that the dielectric properties and the chemical composition of the GTR were modified due to the devulcanization process. This chemical and electromagnetic change due to the devulcanization process is clearly driven by temperature, and critical temperatures between 160 and 195 °C have been identified during permittivity measurements.

This study also shows that the permittivity changes depend on microwave power regimes, in addition to temperature. Consequently, different amounts of energy are required to start the devulcanization process when relying upon the microwave power regime. This knowledge can be utilized to further understand the dielectric behavior of rubber during microwave devulcanization and provides the basis for the improvement and innovation of this sort of devulcanization process.

## 2. Materials and Methods

### 2.1. Materials

The material under test (MUT) was crumb rubber from ELTs (GTR) provided by Synthelast S.A. (Elche, Spain). The GTR had a granule size range of [0.5, 1] mm and an average bulk density of 400 kg·m^−3^. The holder used for the samples was a designed test tube made of quartz. The tube had a cylindrical shape with an inner diameter of 10 mm (outer diameter of 12 mm). It had a height of 120 mm. Nevertheless, the sample inside the test tube was not higher than 15 mm to ensure uniform heating inside the cavity. Thus, each of the six samples had 0.5 g of GTR and a volume of around 1.178 cm^3^.

### 2.2. Characterization of the GTR

A thermogravimetric analysis (TGA) was conducted to determine the composition of the GTR. The analysis was performed with a Mettler Toledo TGA/DSC 1HT thermogravimetric analyzer. The amount of GTR used for the thermal characterization was about 10 mg. To create an inert atmosphere, a constant temperature (30 °C) was kept under a 50 mL·min^−1^ nitrogen flow for the first 30 min. The next step was an increase in that temperature to 800 °C, with a heating rate of 10 °C·min^−1^. Such a temperature was maintained for 15 min. After that, the sample was cooled down to 300 °C, with a cooling rate of 30 °C·min^−1^. During the final step, the sample was heated again at a rate of 20 °C·min^−1^, under 50 mL·min^−1^ of air flow, in order to determine the carbon black content of the sample.

A sulfur analysis was performed with a Leco S 628 sulfur analyzer (St. Joseph, MI, USA) in order to determine the presence of sulfur content in the GTR. The amount of GTR used for this test was about 90 mg.

### 2.3. Measurement Setup for Dynamic Relative Permittivity of GTR Samples

**Figure 1:**
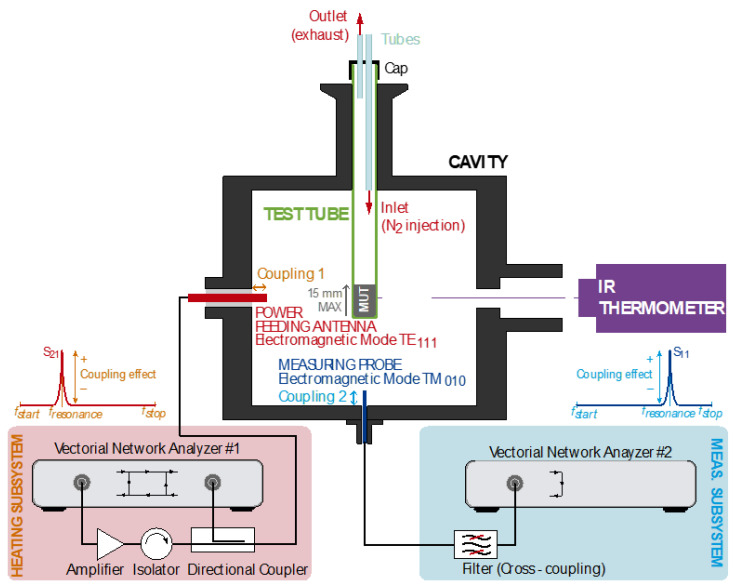
Sketch of the test setup.

A dual-mode cylindrical microwave cavity with dimensions of 104.92 mm in diameter and 85 mm in height was used to simultaneously heat and devulcanize the GTR samples and to measure their complex relative permittivity, operating with transverse electric (TE_111_) and transverse magnetic (TM_010_) modes, respectively [30]. The magnetic field in the TM_010_ electromagnetic field pattern is perpendicular to the propagation direction, leaving only an electric field component in that direction. The power signal for heating the MUT was generated using a two-port Rohde & Schwarz ZVRE vector network analyzer (Munich, Germany) (VNA) and a RCA2026U50 RFcore Ltd. Amplifier (Seongnam-si, Republic of Korea), as depicted in Figure 1, delivering up to 150 W. The smooth control of the delivered power was set by adjusting the level of coupling of the feeding antenna (penetration inside the cavity) which excites the TE_111_ [31], and an adjustable frequency span at the VNA within the [2.2, 2.6] GHz band around the resonance peak. The latter produces an effective duty cycle for the heating, since the sweeping time is linked to the frequency span, and the effective heating happens only when the system is coupled during the sharp resonance. On this premise, the span was restricted to [2.3978, 2.4027] GHz.

The permittivity measurements were accomplished using a second VNA covering the resonance for the TM_010_ mode by sweeping the range [1.9, 2.2] GHz in a period of 1.2 s. The sampling resolution was set to Δf = 2 MHz (150 frequency points).

The extraction of the complex permittivity from the scattering parameters was computed using the cavity perturbational method (CPM) as described in [30], by means of Equations (1) and (2), as follow:

(1) $$\varepsilon^{\prime }=1+\frac{-\frac{\Delta f}{f}\left(\eta +N\frac{\Delta f}{f}\right)-N{\left[\Delta \left(\frac{1}{2Q}\right)\right]}^{2}}{{\left(\eta +N\frac{\Delta f}{f}\right)}^{2}+N^{2}{\left[\Delta \left(\frac{1}{2Q}\right)\right]}^{2}}$$

(2) $$\varepsilon^{\prime \prime }=\frac{\eta \Delta \left(\frac{1}{2Q}\right)}{{\left(\eta +N\frac{\Delta f}{f}\right)}^{2}+N^{2}{\left[\Delta \left(\frac{1}{2Q}\right)\right]}^{2}}$$

where $\frac{\Delta f}{f}$ is the shift in the resonant frequency, $\Delta \left(\frac{1}{2Q}\right)$ is the change in the Q-factor, N, ranging from 0 to 1, is the sample depolarization factor in the direction of the electric field polarization, and *η* is the sample filling factor, as explained in [30]. The estimated accuracy of the dielectric constant is around 3%, and around 10% for the loss factor within the range [0.001, 0.1].

Temperature measurements were performed according to the procedure reported in [32]. Therefore, the temperature for the GTR samples was monitored using a Fluent RF23 infrared thermometer and measured at the tube surface with an accuracy of 0.1 °C. To prevent any combustion from rubber granulates or any flammable gaseous by-products, it was required to ensure an inert atmosphere inside the test tube by enabling an inert gas flow. The gas inlet provided nitrogen to displace the oxygen and any gaseous by-products through the outlet.

### 2.4. Sensible Power and Energy Absorbed in GTR Samples

The microwave heating system measured the power delivered to the cavity through coupling 1 by monitoring both the incident and the reflected power. Moreover, for the evaluation of the provided microwave power’s effectiveness with respect to the sample temperature, other processes should be taken into account as energy scatters. There are thermal losses from the GTR due to thermodynamics (radiation and conduction), but there are also other phenomena, such as the cooling down effect from the nitrogen intake, the enthalpy caused by the chemical reactions (sulfur bonds breaking, generation of sulfur gases), and the mechanical stresses within the granules as the sulfur gases are promoted and released from their cores. Therefore, the time-discretized sensible absorbed power parameter is considered as an indicator of the power related to the effective sample heating by means of the following Equation (3):

(3) $$P_{abs}\left(t_{k}\right)=m_{sample}\cdot c_{p}\cdot \frac{\Delta T_{k}}{\Delta t_{k}}=m_{sample}\cdot c_{p}\cdot \frac{T_{k}-T_{k-1}}{t_{k}-t_{k-1}\;};k\geq 1,$$

where $m_{sample}$ (kg) is the sample mass, $c_{p}$ (J·g^−1^·K^−1^) is the specific heat of GTR, and $\Delta T_{k}$ (°C) is the increase in temperature for the time interval $\Delta t_{k}$ (s). The value of specific heat for GTR samples has been set to 1.9 J·g^−1^·K^−1^ for all calculations [33]. It should be noted that this is only an approximation, because the GTR specific heat varies with temperature and during the devulcanization process [34].

Therefore, the amount of sensible energy absorbed by the MUT as a function of the sensible absorbed power $P_{abs}$ for an irradiation time, and considering the time-discretization of the records, has been estimated in this work as follows:

(4) $$E_{abs}\left(\Delta t_{n}\right)=\sum_{k=1}^{n}\bar{P_{abs}\left(\Delta t_{k}\right)}\cdot \Delta t_{k}=\sum_{k=1}^{n}\frac{P_{abs}\left(t_{k}\right)+P_{abs}\left(t_{k-1}\right)}{2}\cdot \left(t_{k}-t_{k-1}\;\right);\;n\geq 1,$$

where $\Delta t_{n}=t_{n}-t_{0}$ is the irradiation duration ($t_{0}$ is the time when the irradiation started), and $t_{k}$ is the k-*th* sampled time where the temperature $T_{K}$ was recorded to compute $P_{abs}\left(t_{k}\right)$, as in (3).

### 2.5. Characterization of the Rubber Network Structure in GTR Samples

Time-domain NMR experiments were applied to GTR samples to evaluate the evolution of the rubber network structure according to the applied microwave-assisted devulcanization protocol following the procedure and data analysis explained elsewhere [35]. Solid-state ^1^ H double-quantum (DQ) NMR experiments were carried out in a Bruker minispec mq20 spectrometer (Billerica, MA, USA) operating at 0.5 T with 90° pulses of 3.1 µs length and a dead time of 12 µs at a temperature of 80 °C. The recycle delay time was fixed to 0.5 s for filtering the liquid-like NMR signal from processing oils and other small molecules (e.g., accelerators, waxes, and antioxidants) but ensuring the complete magnetization of rubber protons, with a characteristic longitudinal relaxation time of T_1(rubber)_~60 ms [35].

The GTR samples were inserted into NMR glass tubes of 10 mm outer and 8 mm inner diameter, reaching a maximum height of 5 mm to ensure the homogeneity of the magnetic field. Data analysis was based on a previously explained point-by-point normalization process [36] and a subsequent numerical inversion procedure (based on a fast Tikhonov regularization [37]) in order to quantify both the non-coupled fraction of network defects (mostly related to dangling chain ends in GTR samples) and the actual distribution function of residual couplings. The latter is directly related to the cross-linked network structure of rubber, as was previously demonstrated in different rubber samples [38,39,40], including GTR [35].

## 3. Results

The results for the thermogravimetric and sulfur analysis, as well as the records for the GTR temperature curves, the permittivity values versus temperature and sensible absorbed energy, and the microwave power delivered to the cavity are presented in this section for all microwave-assisted devulcanization tests. The estimation of the sensible power and the energy absorbed by the GTR samples during the devulcanization tests has also been carried out to analyze the permittivity evolution versus absorbed energy.

The average permittivity of the GTR samples at the initial state, i.e., before microwave irradiation, was 2.46−j0.055 at 20 °C.

### 3.1. GTR Characterization

The results of the thermogravimetric analysis of GTR are summarized in Table 1. The unburned residue (8.5%) was attributed to inorganic components in the sample, such as minerals and metals. The results of the sulfur analysis showed that the presence of sulfur content in the GTR is 1.9%. The amount of sulfur that has been measured in GTR is in line with the percentages in reference [41].

### 3.2. Temperature Evolution over Time

**Figure 2:**
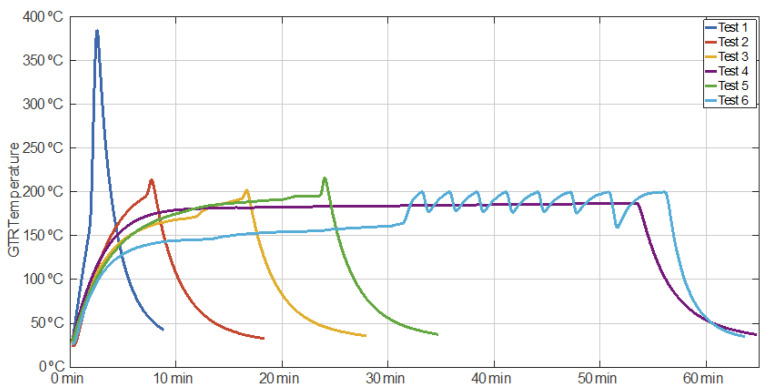
Monitored temperature of the tested samples versus time.

Figure 2 shows the GTR temperature evolution for all tests. For Test 1, the maximum temperature value recorded was 392 °C, and it was reached in 2 min and 54 s with an average heating rate of around 128.3 °C·min^−1^. An important aspect is that this sample was pyrolyzed due to the high temperature and, therefore, it was carbonized.

In Test 2, the temperature grew from 20 °C to 215 °C, and it reached its maximum in approximately 8 min. Thus, the average heating rate was 25 °C per minute.

During Test 3, the sample temperature hit its maximum value of 203 °C in approximately 17 min, Thus, the average heating rate for this case was 10.8 °C·min^−1^.

Regarding the temperature behavior during test 4, it increased from 20 °C to 180 °C in approximately 10 min, which shows a heating rate of 16 °C·min^−1^. Following that, the microwave power was modulated to maintain a temperature range of 180 °C to 187 °C for 45 min.

Concerning Test 5, the sample temperature reached its maximum value (220 °C) in approximately 24 min. As a result, its average heating rate was approximately 8.3 °C·min^−1^.

In Test 6, the sample temperature was incremented from 20 °C to 160 °C in approximately 31 min, meaning an average initial heating rate of around 4.5 °C·min^−1^. Afterwards, a microwave pulsed power sequence was run to cause eight heating-cooling cycles in the sample. Each cycle was completed by heating the sample up to 200 °C and then letting it cool down to approximately 175 °C. An important heating rate of 20 °C·min^−1^ for the first cycle can be observed from minute 31, when the sample had a temperature of around 162 °C.

### 3.3. Power Profiles

**Figure 3:**
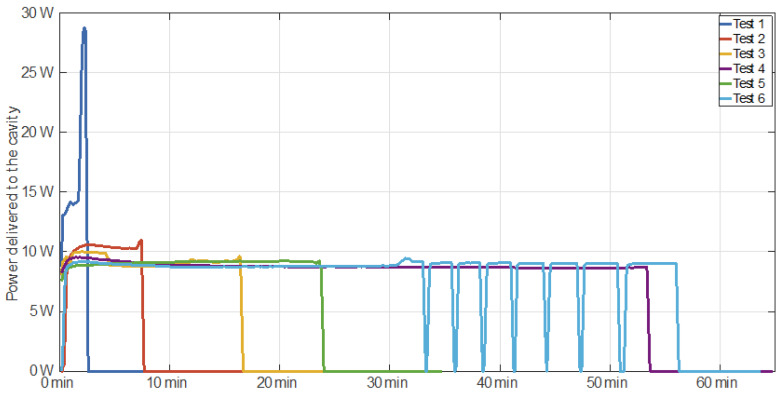
Monitored power delivered to the cavity versus time for each test.

Figure 3 shows the microwave power delivered to the cavity during the different microwave-assisted devulcanization tests. For Test 1, three different periods can be observed in the delivered power profile. During the first period, the power was relatively high, around 14 W, whereas during the second stage, the power drastically increased, up to a maximum value of 29 W. The third period is related to the free cooling of the sample, where no microwave power was applied at all.

For Tests 2, 3, 4, and 5, two different periods can be observed for the power. During the first period of Test 2, a nearly constant power value of 10 W was applied. A constant power of around 9 W was applied during Tests 3, 4, and 5. The second period is related to the free cooling of the sample when microwave power was not delivered at all. It is worth noting that the microwave power was turned off after it was detected that the temperature slope had increased significantly, which resulted in a peak temperature that was considerably lower with regard to Test 1.

For Test 6, three different power profile periods can be observed. The first period is similar to Tests 3, 4, and 5, but with a constant power feed of around 9 W. During the second stage, a sequence of eight on–off power cycles was applied. The third period is related to the sample’s free cooling, so the microwave power was off during this stage.

### 3.4. Permittivity Behavior Versus Temperature

**Figure 4:**
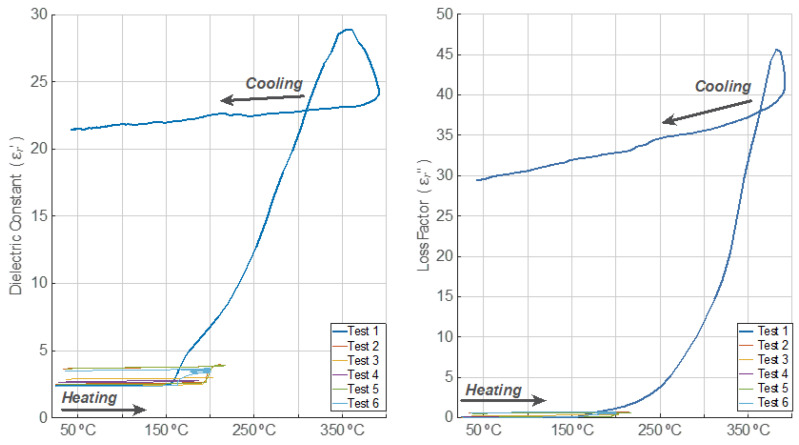
Relative electric permittivity behavior versus temperature for each test.

**Figure 5:**
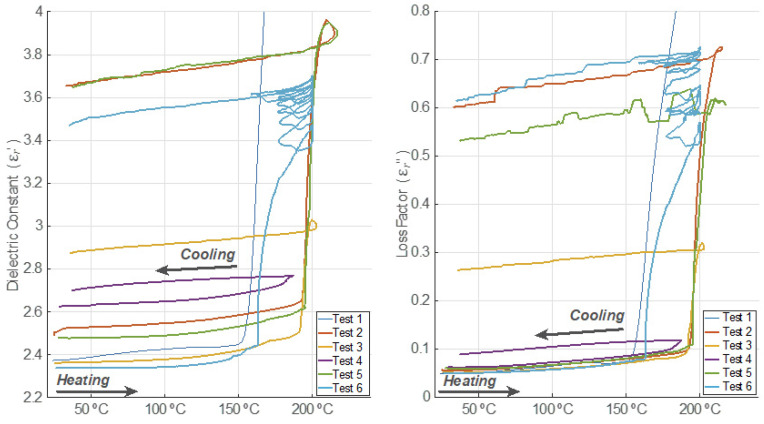
Relative electric permittivity behavior versus temperature for each test (magnified from Figure 4 for a proper visualization of Tests 2 to 6).

In Figure 4 and Figure 5, the dielectric constant and loss factor evolution versus temperature are depicted, respectively. For Test 1, both the dielectric constant and the loss factor show three different dynamics. During the first period, both magnitudes increased their values very slightly. After exceeding the temperature of 160 °C, both magnitudes incremented their values up to maximum levels exponentially. During the third period, which is related to the free cooling of the sample, the dielectric constant and loss factor decrease with decreasing temperatures. However, it can be clearly observed that the values of permittivity are much higher than those measured before the devulcanization process occurs. In fact, the final value of the dielectric constant after the cooling process is, in this case, nine times higher than the initial one. In the case of the loss factor, the final value is 590 times higher than the initial one.

In Tests 2, 3, and 5, three periods are also noticeable. For temperatures lower than 190 °C, both the GTR dielectric constant and the loss factor increased their values slightly. Nevertheless, for temperatures higher than 190 °C, both magnitudes grew in an exponential way until the cooling period started, indicating important chemical changes inside the GTR sample.

In Test 2, the increment of the dielectric constant after the cooling period was around 46.7%, and the loss factor value was multiplied by approximately 10.7 after comparing the final values to the initial ones before the microwave processing. Therefore, it can be observed that, in this case, the permittivity increment after the cooling period is much smaller than the one observed for sample 1.

Regarding Test 3, the increment of the dielectric constant was around 21.7%, and the loss factor value rose five-fold versus the initial values of both magnitudes. Although the final temperature is only slightly lower, these increments are clearly lower than those observed in sample 2.

As can be seen from the results, both the dielectric constant and the loss factor did not increase in Test 4 as much as in previous tests. The dielectric constant rose by around 2.9% after the cooling period, and the loss factor increment was 44.2% when compared to the values before the microwave irradiation. In this test, the sample temperature did not surpass 190 °C.

In Test 5, both the dielectric constant and loss factor behaved similarly to those described for Test 2. The increment of the dielectric constant once the cooling period ended was around 47.2%, and the loss factor multiplied almost nine times compared to its initial value.

Concerning Test 6, both the GTR dielectric constant and loss factor also increased their values with increasing sample temperatures. The dielectric properties varied in the first on–off cycles, but they slowed down to almost constant during the last ones. The final increment of the dielectric constant, after the cooling period, was around 48.3%, and the loss factor was multiplied by 12. In this case, the threshold temperature before steep permittivity increments was detected at around 160 °C.

From the obtained results, it can be clearly deduced that the permittivity does not only depend on temperature but also on microwave power regimes (heating rates) that cause mechanical effects, such as the stress caused by the volumetric expansion of the generated gas within the granule until it escapes away after fissuring the particle, easing the devulcanization process, as can be perceived in Tests 1 and 6. There is a noticeable noise in the loss factor during the cooling period after having triggered the devulcanizing process. This can be explained by the fact that some chemical and mechanical transients might undergo a new steady state. This effect is visible for the loss factor since it is a relative magnitude whose values are less than unity, while the dielectric constant is less sensitive to these effects due to its higher values.

**Figure 6:**
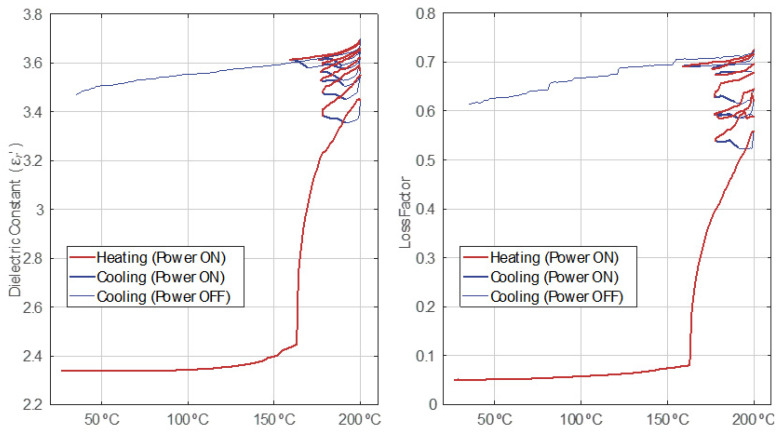
Relative electric permittivity behavior versus temperature with the power on–off cycles discrimination for Test 6.

In Figure 6, the effect of the power on and off cycles on the permittivity behavior versus temperature is emphasized. There is a match between heating cycles with the active irradiation of the sample and the increase in the permittivity levels. Right after every heating cycle, there follows a cooling cycle matching a power off period and a decrease in the dielectric parameters. Surprisingly, with every new irradiation cycle, the sample still shows a cooling down transient before its temperature starts to rise in a new heating cycle, but with a noticeable change in the permittivity tendency slope. This behavior is evidence that microwaves are effectively causing structural changes, while their thermal effects might be masked by endothermic chemical reactions and/or mass losses due to the fumes emitted.

### 3.5. Model for the GTR Dielectric Properties during Devulcanization

Both the dielectric constant and loss factor curves during the heating process have been modelled as a 2-piecewise function described in Equation (5), whose parameters are detailed in Table 2 and Table 3. Equation (5) is as follows:

(5) $$f\left(T\right)=\{\begin{array}{cc} a_{0}T^{\frac{1}{0.3}}+a_{1}T+a_{2} & ;T\in \left[20,\;b_{2}\right]\;℃\\ b_{0}\left(1-e^{-\frac{T-b_{2}}{b_{1}}}\right)+b_{3}\left(T-b_{2}\right)+b_{4} & ;T\in \left[b_{2},\;220\right]\;℃ \end{array}$$

where *T* is the crumb rubber temperature in degrees Celsius.

**Figure 7:**
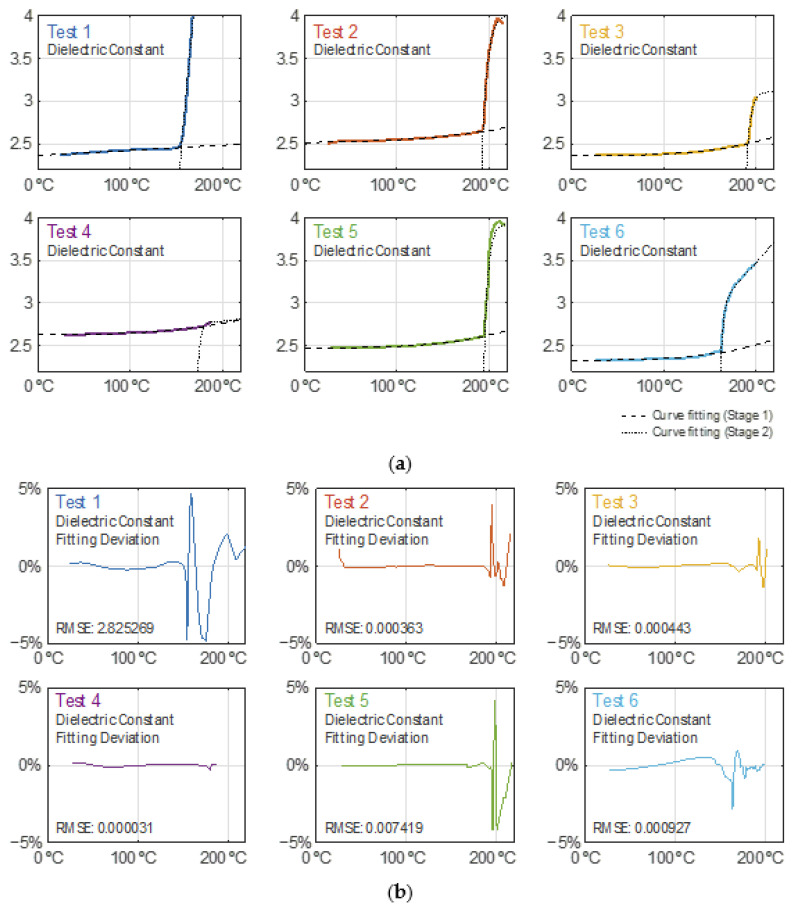
(**a**) Dielectric constant behavior during the heating and curve fitting modelling for each test. (**b**) Dielectric constant curve modelling relative residuals for each test.

**Figure 8:**
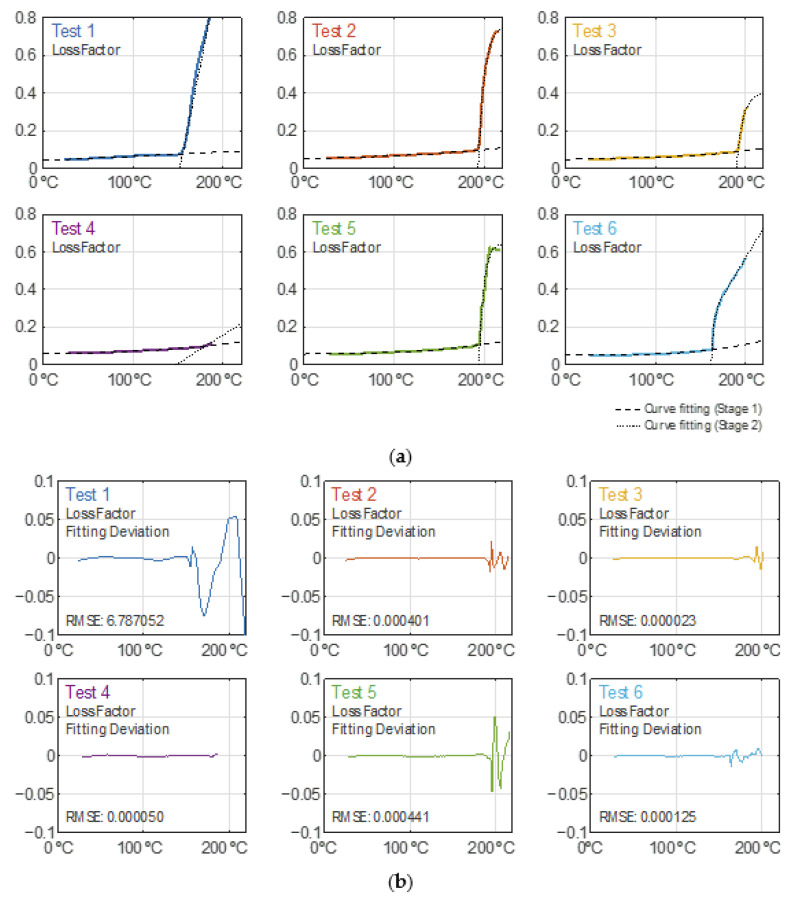
(**a**) Loss factor behavior during the heating and curve fitting modelling for each test. (**b**) Loss factor curve modelling absolute residuals (linear scale) for each test.

In Figure 7a, the dielectric constant data traces and their fitted curves for the heating records are depicted, while Figure 7b shows their corresponding residuals, which are within a ±5% relative deviation. Likewise, Figure 8a,b are related to the modelling of the loss factor, where it is shown to have an absolute deviation of ±0.1 as the boundary. The latter is not presented on a relative scale because of its high sensitivity to very low levels which would distort its interpretation. For Test 6, only the records for its first heating cycle have been considered in this analysis (until minute 31.5).

From the obtained results, one can conclude that both the dielectric constant and loss factor monotonically increase as the sample temperature increases. It is when the temperature overcomes a certain temperature threshold that the dielectric parameters grow significantly. The temperature threshold for each piece (stage) defined by Equation (5) differs for each test due to their particular curve kinks (modeled by the parameter $b_{2}$) as a result of their own microwave irradiation profiles, as noticed in Figure 7a and Figure 8a. The case for the Test 4 curves does not show as sharp a kink as in the other tests.

### 3.6. Speed of Permittivity Changes during Devulcanization

**Figure 9:**
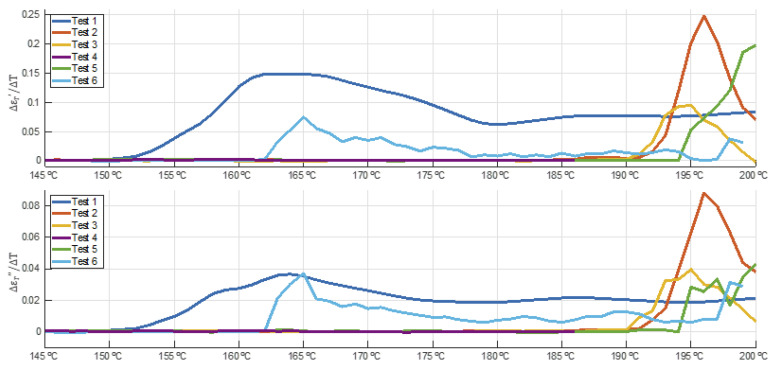
Dielectric constant and loss factor gradients versus temperature (resolution Δ*T* = 1 °C).

For temperatures below 150 °C, the gradient of dielectric constant and loss factor values versus ascending temperature is nearly zero. The interest of this analysis lies within the range between 145 and 200 °C, as shown in Figure 9. These curves show a sharp growth of the dielectric parameters between 190 and 194 °C for tests 2, 3, and 5, while this growth is anticipated at 150 °C and 162 °C for tests 1 and 6, respectively. For Test 6, only the records for its first heating cycle have been considered in this analysis (until minute 31.5).

The difference in the threshold temperatures in Figure 9 might be explained by the differences for each test in heating rates above 150 °C. Table 4 shows a summary of the threshold temperature, the maximum heating rate, and the maximum sensible absorbed power within the temperature range of between 150 and 195 °C. It can be observed from the data that the higher the heating rate and maximum absorbed power above 150 °C, the lower the threshold temperature and, therefore, the devulcanization process occurs at lower temperatures.

### 3.7. Permittivity Evolution Versus Sensible Absorbed Energy

**Figure 10:**
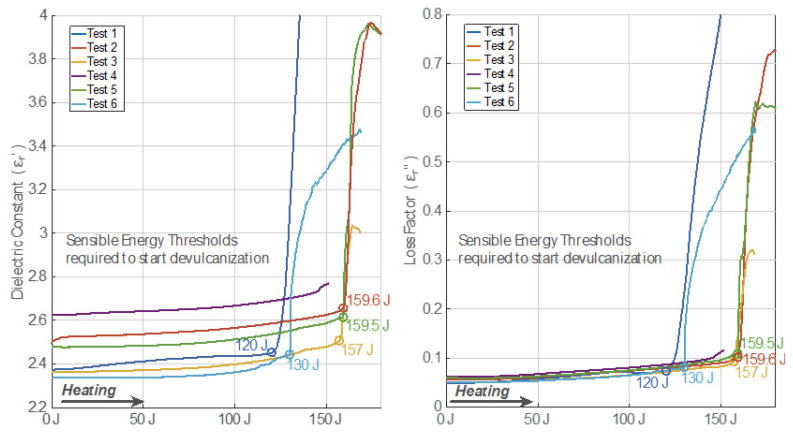
Permittivity behavior versus sensible absorbed energy while heating. Curve kinks are emphasized with a circle showing the energy value at that point.

Figure 10 shows the evolution of both the dielectric constant and the loss factor of crumb rubber samples versus sensible absorbed energy. Energy was calculated by taking into consideration power absorbed over time, as expressed in Equations (1) and (2). Different behaviors are shown depending on both the sample temperature and heating rate at high temperatures (above 150 °C). For instance, the energy threshold for devulcanization in Test 1 is around 120 joules. However, for Test 6, the sample needs 130 J to start the devulcanization. Tests 2, 3, and 5 show a very similar threshold energy of around 160 J. Test 4, with the lowest final temperature and a low heating rate, does not show any threshold evidence.

### 3.8. Evolution of Rubber Network Strcture for GTR Samples during Microwave-Assisted Devulcanization Process

The residual dipolar coupling distribution measured by ^1^ H-DQ NMR experiments (performed in a low-field spectrometer) is directly related to the rubber network structure in terms of the spatial distribution of constraints that increase the dynamic order parameter of the rubber segments [36], e.g., cross-links [38,42], entanglements [43], and rubber interactions at the filler surface [44]. Recently, it has been demonstrated that this experimental approach provides unique information about the structure of devulcanized GTR samples at a molecular level that could address some of the limitations of some other experimental methodologies (mostly based on equilibrium swelling experiments) currently applied in this complex field [35].

**Figure 11:**
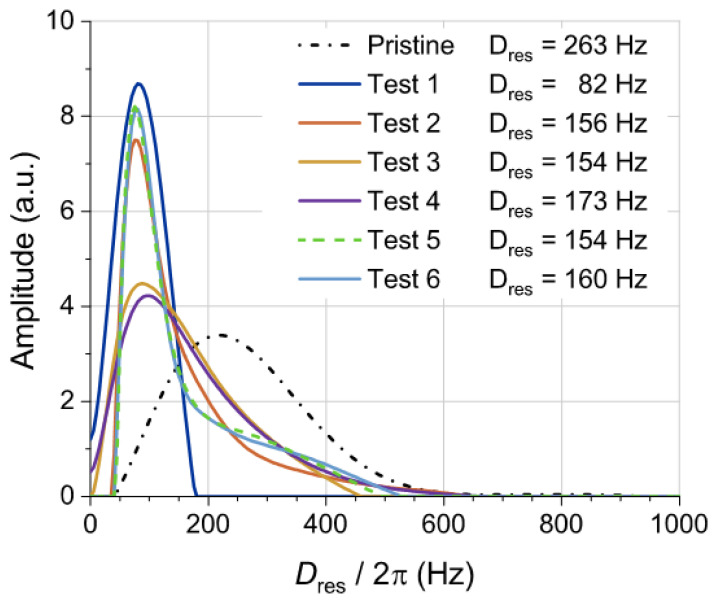
Residual dipolar couplings distributions for pristine GTR and microwave-assisted devulcanized GTR samples. The average (*D*_res_) of each distribution is shown in the legend.

Figure 11 shows the residual dipolar coupling distribution of pristine GTR, defining the density of constraints according to its average value, *D*_res_ = 263 Hz, which is in good agreement with previously reported values for GTR samples from truck tires [35]. The application of microwave treatments, independently of the different test settings, provokes a significant displacement of the distribution toward lower *D*_res_ values, demonstrating the decrease in the cross-link density and the efficiency of each performed test in terms of devulcanization degree. It is important to note that the elastic network structure of devulcanized GTR samples from Test 2, Test 3, Test 5 and Test 6 is quite similar, with *D*_res_ values between 154 and 160 Hz (a decrease of around 100 Hz as compared with the pristine GRT sample). The rubber sample obtained from Test 4 shows a slightly lower devulcanization efficiency (*D*_res_ = 173 Hz), whereas the sample obtained from Test 1 shows quite different behavior in the distribution shape and its average value (*D*_res_ = 82).

The distributions after the microwave treatment are shifted toward lower *D*_res_ values, but some rubber network structure is still measurable because of the persistence of a fraction of unaffected cross-links (mainly monosulfidic cross-links) and entanglements. Additionally, these distributions show some bimodality in the shape. These shoulders in the distributions at higher *D*_res_ values should be related to higher restricted rubber segments at the filler surface because of the adsorbed bound rubber in carbon black or covalently bonded rubber segments in the case of silica modified with bifunctional silanes. This population of rubber at the filler surface is only observable when the overlapping signal from the rubber network structure is shifted to lower cross-link densities after the devulcanization process [35]. It means that the rubber molecular weight between cross-links for the pristine GTR sample is quite similar to the molecular weight between interactions at the filler surface. This is observed in all devulcanized samples except for the specimen obtained after Test 1. In this particular case, the high temperature recorded (392 °C) is able to pyrolyze the rubber around the carbon black particles, eliminating the contribution of rubber–filler interactions to the residual dipolar coupling distribution.

**Figure 12:**
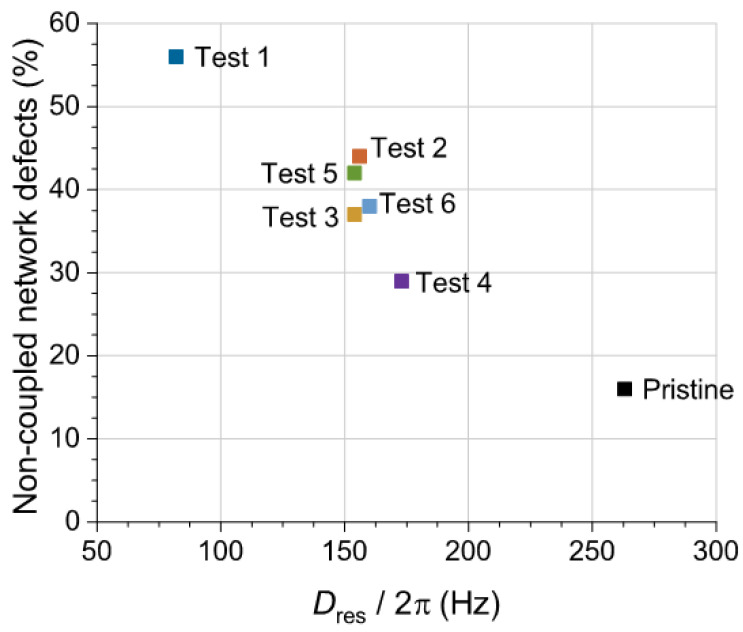
Evolution of the noncoupled defect fraction as a function of the average residual dipolar coupling (proportional to the density of constraints in the rubber matrix) for pristine GTR and microwave-assisted devulcanized GTR samples.

The decrease in the cross-link density of GTR samples after the microwave-assisted devulcanization process promotes the increase in the quantified fraction of non-elastic network defects from less than 20% for the pristine GTR sample to more than 55% for the devulcanized sample after Test 1, as shown in Figure 12. This fraction of network defects is mostly composed of extractable rubber segments (which are not attached to the rubber network) and dangling chain ends. Although the full quantification of these two contributions will require further investigation, it is important to observe the linear correlation with the variation of *D*_res_, which is mostly related to the restrictions imposed by cross-links, entanglements, and filler–rubber interactions in the GTR samples.

## 4. Discussion

In this work, dynamic permittivity measurements for GTR powder during its microwave-assisted devulcanization process were presented. Different temperatures and microwave heating rates were used to determine how the dielectric properties of GTR are affected by those magnitudes. These measurements and the microwave heating processes were carried out with resonant techniques near the 2.45 GHz ISM frequency. The dependence of both the dielectric constant and loss factor on absorbed energy was also analyzed.

The obtained results seem to indicate that temperature level, the energy delivered, and the heating rate (power) have a strong influence on the variation of the GTR dielectric properties, with a set point of 150 °C as the minimum threshold for the devulcanization process.

In Table 5, the initial permittivity of GTR, its permittivity after the irradiation process, the maximum sample temperature during the process, the microwave irradiation time, and the average heating rate are shown for all tests. Some dispersion of the experimental data was observed for the initial permittivity values of GTR. This might be explained as being mainly due to the random distribution of the GTR heterogeneous granules inside the test tube.

As it can be observed in Table 5, the variation in permittivity values is linked to the maximum sample temperature. As a general trend, the higher the maximum temperature, the higher the increase in the dielectric and loss factor values. However, these variations do not rely only on the maximum temperature. Minimum permittivity changes are perceived when the maximum temperature does not reach a certain threshold value, as happens with Test 4. The effective threshold temperature is somehow related to the maximum heating rate observed above 150 °C (see Table 4). Tests 3 and 6 have similar maximum temperatures, but Test 6 ends up with higher permittivity values, and this could be explained by the fact that Test 6 exhibits a higher heating rate above 150 °C and a higher amount of delivered energy.

The average heating rate does not seem to be a relevant factor for the permittivity changes as revealed by Tests 2 and 5 in Table 5. Although Test 2 heats the sample three times faster than Test 5, the final permittivity values are very similar in both cases.

As it can be deduced from Table 5, microwave irradiation time is not a relevant parameter for permittivity changes. Short irradiation times can produce large permittivity increments, provided that the threshold temperature is surpassed.

Concerning the implications that these permittivity measurements have upon the GTR microwave-assisted devulcanization process, several considerations should be taken into account, as follows:

- The devulcanization threshold temperature is within the range of between 150 and 195 °C, and its effective value is conditioned by the irradiation profile;
- Since an exponential growth of permittivity might occur with temperatures above threshold temperatures, microwave power must be controlled beyond this point to avoid thermal runaway effects;
- A uniform heating pattern should be achieved to avoid local hot spots that may lead to dispersed thermal runaways while other payload volumes are not yet enabled to start their devulcanization.

The chemical composition and structure of GTR samples are linked to its effective permittivity. The matrix material of GTR is composed of vulcanized rubber crosslinked by means of poly-, di-, and mono-sulfidic bonds. A certain amount of energy is required to devulcanize the GTR as a total or partial breaking of the sulfur bonds formed during its original vulcanization and/or revulcanization during its granulation process. The energy needed to break the S-S and C-S bonds is 227 kJ/mol and 273 kJ/mol, respectively, while 348 kJ/mol is required for the C-C bonds [45]. The energy to break poly-sulfidic bonds can be as low as 120 kJ/mol [46].

This study has demonstrated that, after applying at least 120 J to the GTR samples (240 J/g), their chemical structure changes due to the devulcanization process as sulfur is released in the form of gas. With a representative specific heat of 1.9 J·g^−1^·K^−1^ and a mass of 0.5 g, it would take 123.5 J to raise its temperature from 20 °C to 150 °C, and 166.2 J to raise it to 195 °C. Assuming the typical percentage of sulfur in GTR to be around 1.9%, it produces 0.095 g (296.27 μmol) of sulfur per sample. Therefore, the estimated energy required to break all the sulfur bonds is within the range of between 2.2 and 58.8 J, depending on the distribution of sulfur link types. Thus, the estimation for the total energy that would be required to heat plus break links is within 129.1 and 182.3 J, or 171.8 and 225.1 J, considering either 150 °C or 195 °C as temperature thresholds. Such estimated lower boundaries are very close to the sensible energy thresholds (from 120 J to 160 J) found in this study for each temperature threshold (deviations of +7.6% and +7.4%).

Despite the conclusions given for the GTR permittivity dynamic behavior versus temperature based on tests performed at around 2 GHz, most of them should be reproducible at lower and higher microwave frequencies where dipolar rotation is still the driving mechanism for the dielectric losses, mainly caused by the carbon black filler.

Time-domain NMR measurements confirm the GTR devulcanization at different degrees since all the microwaved samples show lower residual dipolar couplings (*D*_res_) than pristine GTR. It can be observed that the decrease in *D*_res_ (as measured by NMR) can be correlated with the increase in permittivity values. Therefore, further investigation is envisaged to find the actual relationship between the changes in *D_res_*, the permittivity evolution, and the data provided by additional analysis using differential scanning calorimetry (DSC) or Fourier-transform infrared spectroscopy (FTIR).

The obtained values of the granular GTR permittivity during its microwave-assisted devulcanization show that this applied microwave technology is suitable and very convenient, allowing an optimized process based on the fact that the GTR dielectric properties and the degree of devulcanization seem to have a strong correlation.

## Figures and Tables

**Table 1:** Parameter values from the TGA and DTG curves for GTR.

Component	Percentage
Natural rubber	36.0
Carbon black	27.0
Synthetic rubber	22.0
Inorganic components	8.5
Additives and oil	4.6
Sulfur	1.9

**Table 2:** Fitting curve parameters for the dielectric constant model described by Equation (5).

Test No.	${{\varepsilon^{\prime }}_{r}\left(T\right)|}_{stage\;1}$				${{\varepsilon^{\prime }}_{r}\left(T\right)|}_{stage\;2}$			
	$a_{0}\cdot 10^{-9}$	$a_{1}\cdot 10^{-6}$	$a_{2}$	$b_{0}$	$b_{1}$	$b_{2}$	$b_{3}$	$b_{4}$
1	0	596.7	2.362	1.480	23.350	**154**	0.724	2.260
2	1.990	231.1	2.514	1.488	5.962	**193**	0	2.536
3	3.387	0	2.363	0.707	4.417	**191**	0	2.404
4	2.396	115.5	2.625	0.035	2.780	**181**	0.001	2.734
5	3.147	0	2.476	1.435	4.325	**195**	0	2.485
6	3.794	0	2.330	0.541	4.784	**163**	0.009	2.584

**Table 3:** Fitting curve parameters for the loss factor model described by Equation (5).

Test No.	${{\varepsilon^{\prime \prime }}_{r}\left(T\right)|}_{stage\;1}$				${{\varepsilon^{\prime \prime }}_{r}\left(T\right)|}_{stage\;2}$			
	$a_{0}\cdot 10^{-9}$	$a_{1}\cdot 10^{-6}$	$a_{2}\cdot 10^{-3}$	$b_{0}$	$b_{1}$	$b_{2}$	$b_{3}$	$b_{4}$
1	0	224.5	41.29	0	-	**154**	0.024	0.057
2	0.421	143.6	50.19	0.7281	6.468	**193**	0.001	0.003
3	0.635	82.8	47.19	0.3363	7.185	**191**	0	0.066
4	0.590	124.1	56.61	0	-	**181**	0.003	0.101
5	0.826	64.6	56.31	0.5151	5.410	**195**	0	0.132
6	1.167	17.7	49.66	0.1668	2.523	**163**	0.008	0.106

**Table 4:** Threshold temperature for microwave-assisted devulcanization, maximum heating rate, and sensible absorbed power.

Test No.	Threshold Temperature (°C)	Maximum Heating Rate ^1^ (°C·min^−1^)	Maximum Absorbed Power ^1^ (W)
1	150	372.4	6.15
2	191	27.7	0.38
3	190	22.2	0.35
4	Not Available	18.0	0.22
5	194	12.0	0.16
6	162	42.1	0.63

^1^ between 150 and 195 °C.

**Table 5:** Permittivity before and after test process, maximum temperature, irradiation time, and average heating rate for every test.

Test No.	Initial Permittivity	Permittivity after Test	Maximum Temperature (°C)	Microwave Irradiation Time (min)	Average Heating Rate (°C min^−1^)
1	2.37–0.05 J	21.48–29.40 J	392	2.54	147.0
2	2.49–0.06 J	3.65–0.60 J	215	7.71	25.8
3	2.36–0.05 J	2.87–0.26 J	203	16.67	10.7
4	2.62–0.06 J	2.70–0.09 J	187	53.52	3.0
5	2.48–0.06 J	3.65–0.53 J	217	24.04	7.9
6	2.34–0.05 J	3.47–0.61 J	201	33.22	5.2
